# Musicians show more integrated neural processing of contextually relevant acoustic features

**DOI:** 10.3389/fnins.2022.907540

**Published:** 2022-10-13

**Authors:** Niels Chr. Hansen, Andreas Højlund, Cecilie Møller, Marcus Pearce, Peter Vuust

**Affiliations:** ^1^Aarhus Institute of Advanced Studies, Aarhus University, Aarhus, Denmark; ^2^Department of Clinical Medicine, Center for Music in the Brain, Aarhus University, Royal Academy of Music Aarhus/Aalborg, Aarhus, Denmark; ^3^Department of Dramaturgy and Musicology, School of Communication and Culture, Aarhus University, Aarhus, Denmark; ^4^Department of Linguistics, Cognitive Science, and Semiotics, School of Communication and Culture, Aarhus University, Aarhus, Denmark; ^5^Department of Clinical Medicine, Faculty of Health, Center of Functionally Integrative Neuroscience, Aarhus University, Aarhus, Denmark; ^6^Department of Psychology and Behavioural Sciences, Aarhus University, Aarhus, Denmark; ^7^School of Electronic Engineering and Computer Science, Cognitive Science Research Group and Centre for Digital Music, Queen Mary University of London, London, United Kingdom

**Keywords:** auditory perception, music, expertise, feature integration, mismatch negativity, melody, pitch, magnetoencephalography

## Abstract

Little is known about expertise-related plasticity of neural mechanisms for auditory feature integration. Here, we contrast two diverging hypotheses that musical expertise is associated with more independent or more integrated predictive processing of acoustic features relevant to melody perception. Mismatch negativity (MMNm) was recorded with magnetoencephalography (MEG) from 25 musicians and 25 non-musicians, exposed to interleaved blocks of a complex, melody-like multi-feature paradigm and a simple, oddball control paradigm. In addition to single deviants differing in frequency (F), intensity (I), or perceived location (L), double and triple deviants were included reflecting all possible feature combinations (FI, IL, LF, FIL). Following previous work, early neural processing overlap was approximated in terms of MMNm additivity by comparing empirical MMNms obtained with double and triple deviants to modeled MMNms corresponding to summed constituent single-deviant MMNms. Significantly greater subadditivity was found in musicians compared to non-musicians, specifically for frequency-related deviants in complex, melody-like stimuli. Despite using identical sounds, expertise effects were absent from the simple oddball paradigm. This novel finding supports the integrated processing hypothesis whereby musicians recruit overlapping neural resources facilitating more integrative representations of contextually relevant stimuli such as frequency (perceived as pitch) during melody perception. More generally, these specialized refinements in predictive processing may enable experts to optimally capitalize upon complex, domain-relevant, acoustic cues.

## Introduction

The ability to distinguish and combine features of sensory input guides behavior by enabling humans to engage successfully with perceptual stimuli in their environment (Hommel, 2004; Spence and Frings, 2020). While sophisticated models exist for visual feature processing (Treisman and Gelade, 1980; Nassi and Callaway, 2009; Di Lollo, 2012; Grill-Spector and Weiner, 2014), auditory objects transform over time and remain more elusive (Griffiths and Warren, 2004; Shamma, 2008). Modality-specific divergences may therefore be expected. Indeed, findings that auditory feature conjunctions are processed pre-attentively (Winkler et al., 2005) and faster than single features (Woods et al., 1998) and that the identity features pitch and timbre sometimes take precedence over location (Maybery et al., 2009; Delogu et al., 2014) deviate from findings in visual perception (Campo et al., 2010; but see Guérard et al., 2013). Although auditory feature integration mechanisms are partly congenital (Ruusuvirta, 2001; Ruusuvirta et al., 2003), yet subject to some individual variation (Allen et al., 2017) and evolutionary adaptation (Fay and Popper, 2000), it remains unknown whether they vary with auditory expertise levels. Given its early onset and persistence throughout life (Ericsson, 2006) and its strong reliance on predictive processing mechanisms (Quiroga-Martinez et al., 2021), musicianship offers an especially informative model of auditory expertise (Vuust et al., 2005, 2022; Herdener et al., 2010; Herholz and Zatorre, 2012; Schlaug, 2015).

Electroencephalography (EEG) and magnetoencephalography (MEG) enable approximate estimation of feature integration using the additivity of the mismatch negativity response (MMN) and its magnetic counterpart (MMNm), respectively. The MMN(m) itself represents a deflection in the event-related potential or field (ERP/ERF) peaking around 150–250 ms after presentation of an unexpected stimulus (Näätänen et al., 1978). It results from active cortical prediction rather than from passive synaptic habituation (Wacongne et al., 2012). By comparing *empirical MMN(m)s* to double or triple deviants (differing from standards on two or three features) to *modeled MMN(m)s* obtained by summing the MMN(m) responses for the constituent single deviants, inferences have been made about the potential overlap in neural processing (e.g., Levänen et al., 1993; Paavilainen et al., 2001). Correspondence between empirical and modeled MMN(m)s is interpreted to indicate independent processing whereas subadditivity—where modeled MMN(m)s exceed empirical MMN(m)s—suggests more overlapping, integrated processing. In neurophysiological studies of multisensory integration, this well-established approach has been referred to as “the additive model” relying on the principle that “[b]iophysical laws state that the electrical fields generated by two generators add up linearly at any point measure” (Besle et al., 2009, p. 144). Thus, any observed sub-additivity (or super-additivity, for that matter) would point to an interaction between the two unimodal processes.

Although transferring “the additive model” to a single modality requires additional assumptions in terms of separate neural sources, for example, MMN(m) additivity has successfully been assessed within audition. Segregated feature processing has thus been established for frequency, intensity, onset asynchrony, and duration (Levänen et al., 1993; Schröger, 1995; Paavilainen et al., 2001; Wolff and Schröger, 2001; Paavilainen et al., 2003b) with spatially separate neural sources (Giard et al., 1995; Levänen et al., 1996; Rosburg, 2003; Molholm et al., 2005). MMN(m) is also additive for inter-aural time and intensity differences (Schröger, 1996), phoneme quality and quantity (Ylinen et al., 2005), and attack time and even-harmonic timbral attenuation (Caclin et al., 2006). Feature conjunctions occurring infrequently in the local context, moreover, result in distinct MMN(m) responses (Gomes et al., 1997; Sussman et al., 1998) that are separable in terms of neural sources (Takegata et al., 2001a) and extent of additivity from those elicited by constituent (Takegata et al., 1999) or more abstract pattern deviants (Takegata et al., 2001b).

Conversely, subadditive MMN(m)s occur for combinations of lexical tones, vowels, and consonants in Cantonese speech and non-speech (Choi et al., 2017; Yu et al., 2022), for direction of frequency and intensity changes (Paavilainen et al., 2003a), for aspects of timbre (Caclin et al., 2006), and between frequency deviants in sung stimuli and vowels (Lidji et al., 2009) and consonants (Gao et al., 2012). Generally, subadditivity is greater for triple than double deviants (Paavilainen et al., 2001; Caclin et al., 2006). While it is known that MMN(m) responses vary with expertise (Koelsch et al., 1999; Vuust et al., 2009), it remains unknown whether the same is the case for MMN(m) additivity.

The current MEG study aims to investigate whether MMNm additivity varies as a function of musical expertise. Specifically, assuming that MMNm additivity is a reliable proxy for independent feature processing, two hypotheses are contrasted. First, the *independent processing hypothesis* posits that expertise is associated with specialized feature processing by separate neural populations, increasing access to lower-level representations that have higher context-specific relevance (Ahissar and Hochstein, 2004; Ahissar et al., 2009). This would result in more similar empirical and modeled MMNm responses in musicians compared to non-musicians who, by contrast, would show greater subadditivity. Second, the *integrated processing hypothesis* posits that expertise is associated with processing of multiple features by shared neural resources, manifesting as decreased overall neural activity (Jäncke et al., 2001; Zatorre et al., 2012). This would manifest as smaller empirical than modeled MMNm responses expressed more prominently in musicians than in non-musicians.

Since expertise produces more accurate expectations (Hansen and Pearce, 2014; Hansen et al., 2016) and shorter MMN(m) latencies (Lappe et al., 2016) in musical contexts specifically, these hypotheses will be tested using contrasting paradigms with higher and lower levels of complexity and corresponding musical relevance. Deviants on the three acoustic features frequency, intensity, and location (in terms of inter-aural time difference) will be probed as expertise-related differences in MMN(m) responses to all these particular feature deviants have previously been demonstrated, albeit to different extent and with different levels of selectivity for the type of musical specialization (Nager et al., 2003; Tervaniemi et al., 2006, 2009; Brattico et al., 2009; Putkinen et al., 2014). Because these features represent decreasing degrees of musical relevance—with frequency being syntactically more important than intensity, and intensity being expressively more important than location—their inclusion will allow us to assess feature selectivity for any observed expertise differences in auditory feature integration.

## Materials and methods

### Participants

Twenty-five non-musicians (11 females; mean age: 24.7 years) and 25 musicians (10 females; mean age: 25.0 years) were recruited through the local research participation system and posters at Aarhus University and The Royal Academy of Music Aarhus. Members of the musician group were full-time conservatory students or professional musicians receiving their main income from performing and/or teaching music. The non-musician group had no regular experience playing a musical instrument and had received less than 1 year of musical training beyond mandatory music lessons in school. As shown in Table 1, the two groups were matched on age and sex, and musicians scored significantly higher on all subscales of Goldsmiths Musical Sophistication Index, v. 1.0 (Müllensiefen et al., 2014).

**TABLE 1:** Demographics and musical sophistication of research participants.

	Musicians	Non-musicians	Chi-square test	
			χ *^2^*	*p*
			
Gender	10F, 15M	11F, 14M	0.082	0.774

			Mann–Whitney *U* test	
			
	Mean (SD)	Mean (SD)	*U*	*p*
Age	25.0 (3.9)	24.7 (2.9)	302.5	0.845
GMSI1: Active engagement	49.7 (5.3)	28.3 (10.6)	26.5	< 0.001
GMSI2: Perceptual abilities	55.3 (4.5)	37.7 (6.7)	12.5	< 0.001
GMSI3: Musical training	42.4 (3.1)	10.9 (3.2)	0.0	< 0.001
GMSI4: Emotions	36.2 (4.0)	27.7 (5.0)	61.0	< 0.001
GMSI5: Singing abilities	38.9 (5.1)	21.2 (5.7)	4.0	< 0.001
GMSI6: General sophistication	104.9 (7.3)	49.4 (11.1)	0.0	< 0.001

GMSI 1–6 designate the six subscales of Goldsmiths Musical Sophistication Index v1.0 (Müllensiefen et al., 2014).

All participants were right-handed with no history of hearing difficulties. Informed written consent was provided, and participants received a taxable compensation of DKK 300. The study was approved by The Central Denmark Regional Committee on Health Research Ethics (case 1-10-72-11-15).

### Stimuli

Stimuli for the experiment were constructed from standard and deviant tones derived from Wizoo Acoustic Piano samples from Halion in Cubase 7 (Steinberg Media Technologies GmbH) with a 200 ms duration including a 5 ms rise time and 10 ms fall time.

Deviants differed from standards on one or more of three acoustic features: fundamental frequency (in Hz), sound intensity (in *dB*), and inter-aural time difference (in μ*s*), henceforth referred to as *frequency* (F), *intensity* (I), and *location* (L), respectively. There are several reasons for focusing on these specific features. First, unlike alternative features such as duration (Czigler and Winkler, 1996) and frequency slide (Winkler et al., 1998), the point of deviance can be established unambiguously for frequency, intensity, and location deviants. Second, these features have reliably evoked additive MMN(m) responses in previous research; specifically, additivity has been demonstrated for frequency and intensity (e.g., Paavilainen et al., 2001) and frequency and location (e.g., Schröger, 1995), but not yet for location and intensity or for all three features together. Third, expertise-related selectivity for some of these features over others has been demonstrated in other contexts (Symons and Tierney, 2021). Finally, the restriction to three features balances representability and generalizability with practical feasibility within the typical timeframe of an MEG experiment.

In total, seven deviant types, comprising three single deviants, three double deviants, and one triple deviant, were generated through modification in Adobe Audition v. 3.0 (Adobe Systems Inc.). Specifically, frequency deviants (F) were shifted down 35 cents using the Stretch function configured to “high precision.” Intensity deviants (I) were decreased by 12 dB in both left and right channels using the Amplify function. Location deviants (L) resulted from delaying the right stereo track by 200μs compared to the left one. These parameter values were found to produce robust and relatively similar ERP amplitudes in a previous EEG study (Vuust et al., 2016). Double and triple deviants combining deviants in *frequency and intensity* (FI), *intensity and location* (IL), *location and frequency* (LF), and *frequency and intensity and location* (FIL) were generated by applying two or three of the operations just described, always in the order of frequency, intensity, and location.

### Procedure

**FIGURE 1:**
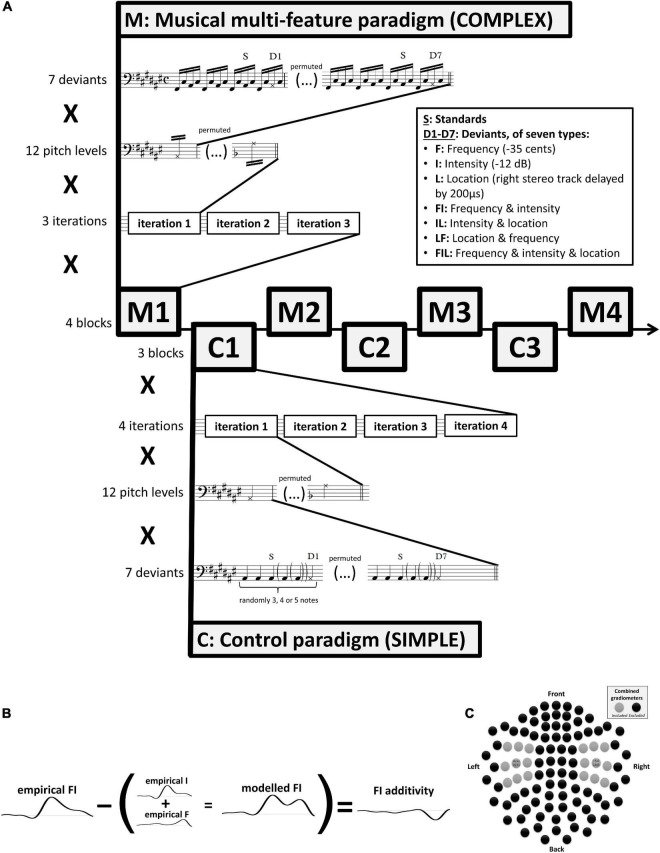
**(A)** Experimental procedure. Stimuli were presented in alternating blocks using the complex musical multi-feature paradigm (M1–M4) and the simple control paradigm (C1–C3). Each block comprised three (M) or four (C) iterations of a sequence of standards and deviants presented at twelve varying pitch levels (i.e., chromatic range of A#2–A3). At each pitch level, seven deviant types were used in permutation. These comprised three single deviants differing in frequency (-35 cents), intensity (-12 dB), or location (right stereo track delayed by 200 μs), double and triple deviants differing in frequency and intensity, intensity and location, location and frequency, or frequency and intensity and location. Epoching of the magnetoencephalographic recordings was time-locked to the onset of the musical notes marked with S for standards and D for deviants. **(B)** Additivity analysis. The additivity of neural mismatch responses was computed by subtracting modeled MMNms—corresponding to the sum of the two or three constituent single-deviant MMNms—from the empirical MMNm obtained in response to the relevant double or triple deviant. **(C)** The 18 combined gradiometers from the Elekta Neuromag TRIUX system that were used in analysis centered on the two sensors—MEG1342 + 1343 and MEG0232 + 0233—exhibiting the peak grand-average MMNm amplitude across both paradigms and across all participants and conditions.

Two MMN paradigms were used in the experiment. Specifically, as shown in Figure 1A, four blocks (M1–M4) of the main complex paradigm, based on the musical multi-feature paradigm (cf., Vuust et al., 2011), were interleaved with three blocks (C1–C3) of a simpler control paradigm (cf., Näätänen et al., 2004) with lower degrees of musical relevance.

The complex paradigm consists of repetitions of a characteristic four-note pattern referred to as the Alberti bass. In this pattern, the notes of a chord are arpeggiated in the order “lowest-highest-middle-highest” (Figure 1A). Although named after an Italian composer who used it extensively in early 18th-century keyboard accompaniment, the Alberti bass occurs widely across historical periods, instruments, and musical genres (Fuller, 2015).

The studies introducing this paradigm (Vuust et al., 2011, 2016, 2012a,b; Timm et al., 2014; Petersen et al., 2015) have typically modified every second occurrence of the third note in the pattern (termed “middle” above) by changing its frequency, intensity, perceived location, timbre, timing, or by introducing frequency slides. In the present implementation, two extra occurrences of the standard pattern were introduced between each deviant pattern (Figure 1A) to minimize spill-over effects from consecutive deviants some of which made use of the same constituent deviant types due to the inclusion of double and triple deviants. Independence of deviant types is an underlying assumption of multi-feature mismatch negativity paradigms (Näätänen et al., 2004).

Consistent with previous studies, individual notes of the pattern were presented with a constant stimulus onset asynchrony (SOA) of 205 ms. After each occurrence of all seven deviants, the pitch height of the pattern changed pseudo-randomly across all 12 notes of the chromatic scale. This resulted in standard tones with frequency values ranging from 116.54 Hz (A#2) to 220.00 Hz (A3). Each block of the musical multi-feature paradigm comprised three such iterations of the 12 chromatic notes (Figure 1A), resulting in a total of 144 trials of each deviant type across the four blocks.

The same number of trials per deviant type was obtained across the three blocks of the simple control paradigm, inspired by Näätänen et al. (2004). This was achieved by incorporating four rather than three iterations of the 12 chromatic notes (A#2 to A3) for each deviant array in each block (Figure 1A). Instead of the Alberti bass, the simple paradigm used standard and deviant notes presented with a constant SOA of 400 ms, corresponding to a classical oddball paradigm. This was a multi-feature paradigm in the sense that it incorporated all seven deviant types in each block; however, the number of standards presented before each deviant varied randomly between 3 and 5, again to minimize spill-over effects from consecutive deviants with the same constituent deviant types (Figure 1A).

During the complete experimental procedure (∼100 mins), participants were seated in a magnetically shielded room watching a silent movie with the soundtrack muted. Stimulus sounds were presented binaurally through Etymotic ER2 insert earphones using the Presentation software (Neurobehavioral Systems, San Francisco, USA). Sound pressure level was set to 50 dB above individual hearing threshold as determined by a staircase procedure implemented in PsychoPy (Peirce, 2007). Participants were instructed to stay still while the sounds were playing, to ignore them, and to focus on the movie. Complex musical multi-feature blocks (M1–M4) lasted 13 mins 47 sec whereas simple control blocks (C1–C3) lasted ∼11 mins. Between each block, short breaks of ∼1–2 mins were provided during which participants could stretch and move slightly while staying seated. Prior to the lab session, participants completed an online questionnaire that ensured eligibility and assessed their level of musical experience using Goldsmiths Musical Sophistication Index, v.1.0 (Müllensiefen et al., 2014).

### Magnetoencephalography recording and pre-processing

MEG data were sampled at 1,000 Hz (with a passband of 0.03–330 Hz) using the Elekta Neuromag TRIUX system hosted by the MINDLab Core Experimental Facility at Aarhus University Hospital. This MEG system contains 102 magnetometers and 204 planar gradiometers. Head position was recorded continuously throughout the experimental session using four head position indicator coils (cHPI). Additionally, vertical and horizontal EOG as well as ECG recordings were obtained using bipolar surface electrodes positioned above and below the right eye, at the outer canthi of both eyes, and on the left clavicle and right rib.

Data were pre-processed using the temporal extension of the signal space separation (tSSS) technique (Taulu et al., 2004; Taulu and Simola, 2006) implemented in Elekta’s MaxFilter software (Version 2.2.15). This included head movement compensation using cHPI, removing noise from electromagnetic sources outside the head, and down-sampling by a factor of 4–250 Hz. EOG and ECG artifacts were removed with independent component analysis (ICA) using the find_bads_eog and find_bads_ecg algorithms in MNE Python (Gramfort et al., 2013, 2014). These algorithms detect artifactual components based on either the Pearson correlation between the identified ICA components and the EOG/ECG channels (for EOG) or the significance value from a Kuiper’s test using cross-trial phase statistics (Dammers et al., 2008; for ECG). Topographies and averaged epochs for the EOG- and ECG-related components were visually inspected for all participants to ensure the validity of rejected components.

Further pre-processing and statistical analysis was performed in FieldTrip (Oostenveld et al., 2011^1^; RRID:SCR_004849). Data were epoched into trials of 500 ms duration including a 100 ms pre-stimulus interval. As indicated in Figure 1A, for both paradigms, only the third occurrence of the standard tone after each deviant was included as a standard trial. Trials containing SQUID jumps were discarded using automatic artifact rejection with a *z*-value cutoff of 30. The remaining 98.2% of trials on average (ranging from 91.0 to 99.8% for individual participants) were band-pass filtered at 1–40 Hz using a two-pass Butterworth filter (data-padded to 3 s to avoid filter artifacts). Planar gradiometer pairs were combined by taking the root-mean-square of the two gradients at each sensor, resulting in a single positive value. Baseline correction was performed based on the 50 ms pre-stimulus interval.

### Experimental design and statistical analysis

To reiterate the experimental design, 25 musicians and 25 non-musicians completed a total of seven blocks distributed between the complex musical multi-feature paradigm (M) and simple control paradigm (C) in the order M1–C1–M2–C2–M3–C3–M4 (Figure 1A). Trials were averaged for each condition (i.e., one standard and seven deviant types) separately for each participant and separately for each paradigm. Magnetic mismatch negativity responses (MMNm) were computed by subtracting the same average standard (the third after each deviant, cf. Figure 1A) originating from the relevant paradigm from each of the deviant responses. These were the empirical MMNms. Consistent with previous studies (Levänen et al., 1993; Schröger, 1995, 1996; Takegata et al., 1999; Paavilainen et al., 2001, 2003b; Ylinen et al., 2005; Caclin et al., 2006; Lidji et al., 2009; Choi et al., 2017; Yu et al., 2022), modeled MMNms were computed for the three double deviants and one triple deviant by adding the two or three empirical MMNms obtained from the relevant single deviants (Figure 1B).

The statistical analysis reported here focused on data from the combined planar gradiometers. Our focus was on the modulation of the neural signals as indexed by the MMNm response. Seeing that previous research has shown little to no added benefit in signal-to-noise ratio for source-derived estimates of the MMN(m) response, likely because the tangentially-oriented MMNm sources are optimally detected by the planar gradiometers (Tervaniemi et al., 2005; Recasens and Uhlhaas, 2017), we deemed that our research question could be fully addressed in sensor space without making further assumptions as required for source reconstruction. Non-parametric, cluster-based permutation statistics (Maris and Oostenveld, 2007) were used to test the prediction that the additivity of the MMNm response would differ between musicians and non-musicians. Because violation of the additive model is assessed through demonstration of a difference between empirical and modeled MMNm responses, this hypothesis predicts an interaction effect between degree of additivity and musical expertise. This was tested by running cluster-based permutation tests on the difference between empirical and modeled MMNm responses, comparing between musicians and non-musicians. This approach for testing interaction effects within the non-parametric permutation framework is advocated by FieldTrip.^2^

The test statistic was computed in the following way: Independent-samples *t*-statistics were computed for all timepoint-by-sensor samples. Samples that were neighbors in time and/or space and exceeded the pre-determined alpha level of 0.05 were included in the same cluster. Despite its similarity to uncorrected mass-univariate testing, this step does not represent hypothesis testing, but only serves the purpose of cluster definition. To this end, a neighborhood structure was generated that defined which sensors were considered neighbors based on the linear distance between sensors. Only assemblies containing minimum two neighbor sensors were regarded as clusters. A cluster-level statistic was computed by summing the *t*-statistics within each cluster and taking the maximum value of these summed *t*-statistics. This process was subsequently repeated for 10,000 random permutations of the group labels (musicians vs. non-musicians) giving rise to a Monte Carlo approximation of the null distribution. The final *p*-value resulted from comparing the initial test statistic with this distribution. Bonferroni-correction was applied to the two-sided alpha level to correct for the four comparisons of modeled and empirical double and triple MMNms, resulting in an alpha level of α = 0.025/4 = 0.00625. When significant additivity-by-expertise interactions were discovered, simple effects of additivity were assessed for musicians and non-musicians separately.

Previous research shows that the MMNm usually peaks in the 150–250 ms post-stimulus time range and is maximally detected with gradiometers bilaterally at supratemporal sites (Levänen et al., 1996; Näätänen et al., 2007). This was confirmed for the current dataset by computing the grand-average MMNm across all participants, all conditions, and both paradigms. This grand-average MMNm peaked in the combined gradiometer sensors MEG1342 + 1343 (right) and MEG0232 + 0233 (left) at ∼156 ms post-stimulus (with secondary peaks extending into the 200–300 ms range). However, to account for possible differences in peak latency and source location between participants and between the various deviant types, the analysis was extended to the 100–300 ms post-stimulus interval and to also include the eight neighboring sensors around the peak sensor in each hemisphere (Figure 1C). The final 2 × 9 sensors were located approximately over the superior temporal lobe in each hemisphere. In this way, prior knowledge was incorporated to increase the sensitivity of the statistical tests without compromising their validity (Maris and Oostenveld, 2007). In that respect, it should be noted that the cluster-based permutation framework does not allow inferences on the onset or offset of specific effects nor of their exact spatial distribution (Sassenhagen and Draschkow, 2019).

Before the main analysis, however, two sets of tests were conducted to ensure the validity of the main analysis. To this end, cluster-based permutation tests were run using the parameters, sensors, and time window specified above (except for the permuted labels, which were changed according to the contrast of interest). First, the difference between standard and deviant responses was assessed to determine whether MMNm effects were present, i.e., whether standard and deviant responses differed significantly in the 100–300 ms post-stimulus range. These analyses were carried out for each deviant type separately for musicians and non-musicians and separately for the two paradigms. Second, to establish that the possible MMNm effects were potentially additive, nine pairwise comparisons were conducted between relevant single and double deviants as well as between relevant double and triple deviants (i.e., FI-FIL, IL-FIL, LF-FIL, F-FI, I-FI, I-IL, L-IL, L-LF, F-LF). This was done across all participants regardless of expertise level. No Bonferroni-correction was applied to these secondary validity checks.

## Results

To anticipate our results, significantly greater MMNm subadditivity was found in musicians compared to non-musicians for frequency-related features in the complex musical multi-feature paradigm. These expertise effects were absent from the simple control paradigm. Further details are reported separately for the two paradigms below.

### Complex musical multi-feature paradigm

In the main experimental paradigm—the complex musical multi-feature paradigm—all seven single, double, and triple deviants elicited significantly larger responses than the standards for musicians as well as for non-musicians (Table 2), indicative of significant MMNm responses in all conditions of this paradigm. Moreover, the triple deviant resulted in a significantly larger MMNm than each of the double deviants (Table 3). Except for a single comparison between the location deviant and the double deviant combining location and frequency, MMNms to all double deviants were significantly larger than MMNms to single deviants, indicating that the addition of an extra feature significantly increased the MMNm amplitude.

**TABLE 2:** Magnetic mismatch negativity response (MMNm).

	Complex musical multi-feature paradigm		Simple control paradigm	
		
	Musicians	Non-musicians	Musicians	Non-musicians
Frequency (F)	0.0002***	0.0002***	0.0003***	0.0014**
Intensity (I)	< 0.0001***	< 0.0001***	< 0.0001***	< 0.0001***
Location (L)	< 0.0001***	< 0.0001***	< 0.0001***	< 0.0001***
FI	< 0.0001***	< 0.0001***	< 0.0001***	0.0003***
IL	< 0.0001***	< 0.0001***	< 0.0001***	< 0.0001***
LF	< 0.0001***	< 0.0001***	< 0.0001***	< 0.0001***
FIL	< 0.0001***	< 0.0001***	< 0.0001***	< 0.0001***

Monte Carlo *p*-values from non-parametric, cluster-based permutation tests of deviant > standard. **p < 0.0050; ***p < 0.0005 (uncorrected alpha). Analysis was conducted on the 100–300 ms post-stimulus time window on nine supra-temporal combined gradiometer sensors in each hemisphere using the maxsum test statistic over 10,000 random permutations.

**TABLE 3:** Potential additivity of the magnetic mismatch negativity response (MMNm) response.

	Complex musical multi-feature paradigm	Simple control paradigm
FIL > FI	< 0.0001***	< 0.0001***
FIL > IL	0.0033**	0.0003***
FIL > LF	< 0.0001***	< 0.0001***
FI > F	< 0.0001***	0.0003***
FI > I	0.0203*	0.0005***
IL > I	< 0.0001***	< 0.0001***
IL > L	< 0.0001***	< 0.0001***
LF > L	n.s.	0.0002***
LF > F	< 0.0001***	0.0093*

Monte Carlo *p*-values from non-parametric, cluster-based permutation tests of triple > double > single deviants. F: Frequency; I: Intensity; L: Location; *p < 0.0250; **p < 0.0050; ***p < 0.0005 (uncorrected alpha). Analysis was conducted on the 100–300 ms post-stimulus time window on nine supratemporal combined gradiometer sensors in each hemisphere using the maxsum test statistic over 10,000 random permutations.

**FIGURE 2:**
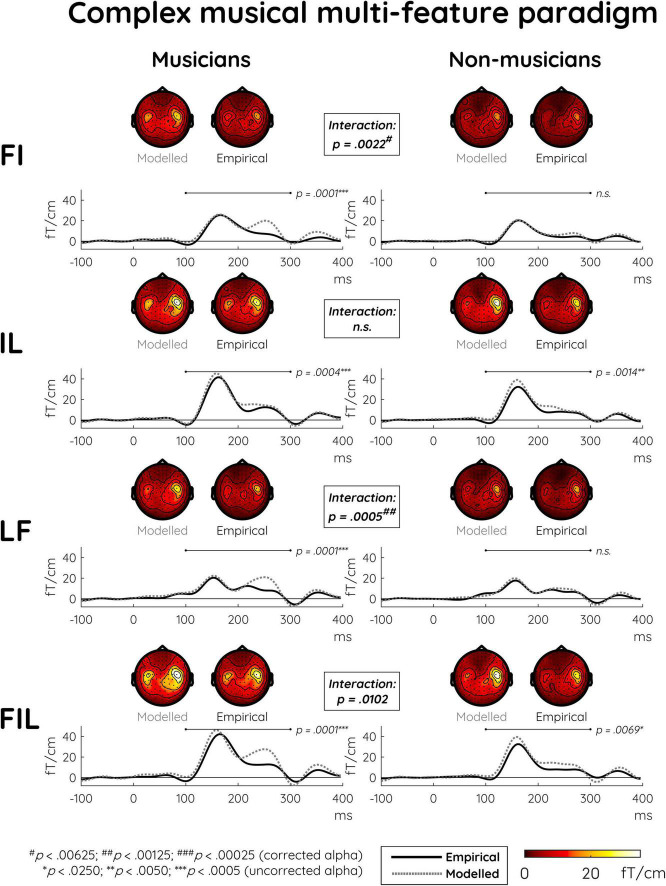
Modeled and empirical mismatch negativity (MMNm) in musicians and non-musicians in the complex musical multi-feature paradigm in response to deviants differing in either frequency and intensity (FI), intensity and location (IL), location and frequency (LF), or frequency and intensity and location (FIL). Modeled MMNms (dashed gray lines) correspond to the sum of the MMNms to two or three single deviants whereas empirical MMNms (solid black lines) correspond to MMNms obtained for double and triple deviants. Comparing the plots for modeled and empirical MMNms, significantly greater subadditivity was evident in musicians compared to non-musicians specifically in the double deviants involving frequency (i.e., FI and LF). This expertise-by-additivity effect was marginally non-significant for the triple deviant (i.e., FIL). The event-related field (ERF) plots depict the mean of the data from the peak sensor and eight surrounding sensors in each hemisphere [the peak gradiometer pairs were MEG1342 + 1343 **(Right)** and MEG0242 + 0243 **(Left)**]. Low-pass filtering at 20 Hz was applied for visualization purposes only. The topographical distributions depict the 100–300 ms post-stimulus time range for the difference waves between standard and deviant responses, and *p*-values outside the boxes reflect simple effects of additivity separately for musicians and non-musicians.

The non-significant LF vs. L comparison as well as the somewhat larger p-values for the comparisons between responses elicited with and without the frequency component (i.e., FIL vs. IL, FI vs. I) already indicated that a certain extent of subadditivity was present specifically for the frequency component. This is also suggested by the dissimilarity between modeled and empirical responses in the first, third, and fourth rows of Figure 2. The subsequent main analysis addressed how this possible effect interacted with musical expertise (see Supplementary Figure 1 for depictions of modeled MMNm responses together with their constituent single-deviant MMNms).

Indeed, the differences between modeled and empirical responses were significantly greater in musicians than in non-musicians for the FI and LF deviants (Table 4). This demonstrated subadditivity was consistent with the *integrated processing hypothesis* as formulated above. For the triple deviant (FIL), this interaction effect approached significance whereas it was absent for the IL deviant, which did not include the frequency component. Consistent with this picture, follow-up analyses of the significant interactions found simple additivity effects only for musicians (Table 4 and Figure 2).

**TABLE 4:** Subadditivity of the magnetic mismatch negativity response (MMNm) response.

	Complex musical multi-feature paradigm			Simple control paradigm		

	(a) Additivity-by-Expertise	(b) Subadditivity: Modeled > Empirical		(a) Additivity-by-Expertise	(b) Subadditivity: Modeled > Empirical	
				
		*Musicians*	*Non-musicians*		*Musicians*	*Non-musicians*
FI	0.0022^#^	<0.0001***	n.s.	n.s.	(0.0027)	(n.s.)
IL	n.s.	(0.0004)	(0.0014)	n.s.	(n.s.)	(0.0005)
LF	0.0005^##^	<0.0001***	n.s.	n.s.	(0.0016)	(n.s.)
FIL	0.0102	(<0.0001)	(<0.0069)	n.s.	(0.0053)	(n.s.)

Monte Carlo *p*-values from non-parametric, cluster-based permutation tests of (a) the interaction of additivity-by-expertise, and (b) simple effects of additivity for musicians and non-musicians. F: Frequency; I: Intensity; L: Location; ^#^*p* < 0.00625, ^##^*p* < 0.00125, (corrected alpha); ***p < 0.0005 (uncorrected alpha). Analysis was conducted on the 100–300 ms post-stimulus time window on nine supratemporal combined gradiometer sensors in each hemisphere using the maxsum test statistic over 10,000 random permutations. *p*-values in brackets are included for completeness but are not relevant due to statistically non-significant interaction effects.

### Simple control paradigm

**FIGURE 3:**
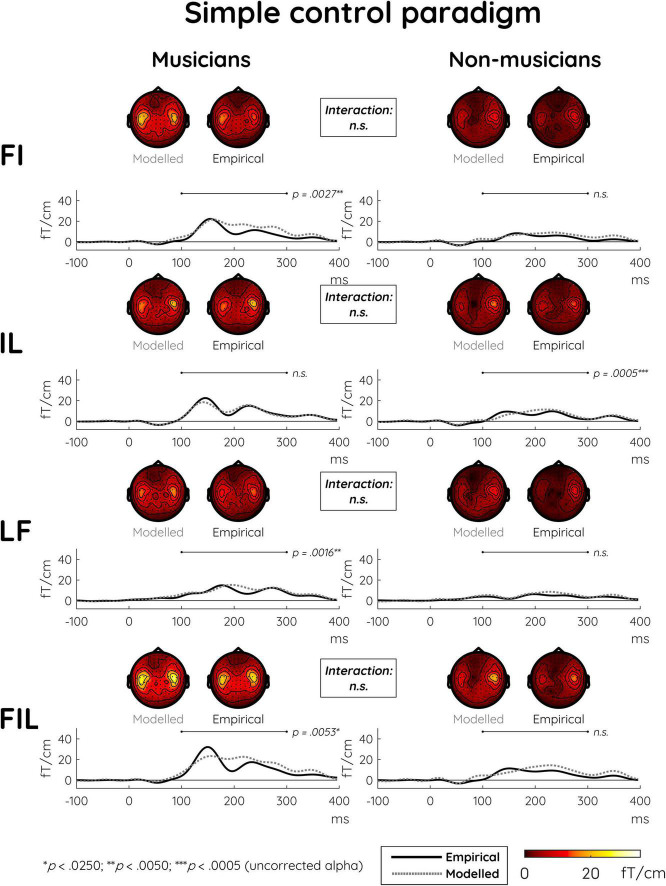
Modeled and empirical mismatch negativity (MMNm) in musicians and non-musicians in the simple control paradigm in response to double deviants differing in either frequency and intensity (FI), intensity and location (IL), or location and frequency (LF), as well as to triple deviants differing in frequency and intensity and location (FIL). Modeled MMNms (dashed gray lines) correspond to the sum of MMNms to two or three single deviants whereas empirical MMNms (solid black lines) correspond to the actual MMNms obtained with double and triple deviants. In contrast to the musical multi-feature paradigm, there were no significant differences in additivity between musicians and non-musicians when comparing the modeled and empirical MMNms. The event-related field (ERF) plots depict the mean of the data from the peak sensor and eight surrounding sensors in each hemisphere [the peak gradiometer pairs were MEG1342 + 1343 **(Right)** and MEG0242 + 0243 **(Left)**]. Low-pass filtering at 20 Hz was applied for visualization purposes only. The topographical distributions depict the 100–300 ms post-stimulus time range for the difference waves between standard and deviant responses, and *p*-values reflect simple effects of additivity separately for musicians and non-musicians.

In the simple control paradigm, significant MMNm effects were also present for all deviant types (Table 2). Here, additivity was more uniformly present in terms of significant differences between all single and double deviants as well as between the double deviants and the triple deviant (Table 3, see Supplementary Figure 2 for depictions of modeled MMNm responses together with their constituent single-deviant MMNms). The main analysis showed no differences in additivity between the two groups and across the various deviant types (Table 4). This pattern also emerges from the plots of event-related fields and scalp topographies in Figure 3.

## Discussion

### Expertise as integrated feature processing

The current results provide the first neurophysiological evidence consistent with more integrated processing of multiple auditory feature deviants in musical experts compared to non-experts. In a complex musical paradigm, magnetic mismatch negativity responses (MMNm) elicited by double deviants differing in multiple features (including frequency) were lower than the sum of MMNm responses to the constituent single-feature deviants. This selective subadditivity for the musically relevant frequency feature was either absent or present to a smaller extent in non-musicians, despite significant MMNms in all conditions. In addition to the main finding of expertise-related differences in frequency-related subadditivity, the pattern of results between the two paradigms suggests that these differences were context-specific. Specifically, the expertise differences were absent when using identical sounds in a simpler and less musically-relevant configuration as a control paradigm.

Following the additive model used extensively in neurophysiological studies of multisensory interactions (Besle et al., 2009) and adapted for studies of unimodal feature integration (e.g., Levänen et al., 1993; Paavilainen et al., 2001), we interpret these findings as support for the integrated processing hypothesis by which musical expertise is associated with enhanced processing by recruiting shared neural resources for more complex representations of domain-relevant stimuli. Importantly, our cross-sectional study design does not allow us to determine whether musical training causally induces more holistic processing or whether pre-existing integration in processing benefits musicianship. A causal interpretation, however, would contrast with formulations of feature integration theory regarding feature processing as innate or acquired through normal neurodevelopment and therefore largely immutable (Treisman and Gelade, 1980; Quinlan, 2003). Findings that perceptual learning modulates attention, thus determining whether specific features are considered for binding (Colzato et al., 2006), have already questioned this view. Indeed, Neuhaus and Knösche (2008) demonstrated more integrative pitch and rhythm processing in musicians than non-musicians manifested in expertise-related differences in the P1 and P2 components. By extending these findings to intensity and location and relating them to MMNm responses, we consolidate the association of musical training with changes in predictive neural processing (Skoe and Kraus, 2012).

Evidence for expertise-related effects on multimodal integration of audiovisual and audiomotor stimuli has steadily accumulated (Paraskevopoulos et al., 2012, 2014; Paraskevopoulos and Herholz, 2013; Bishop and Goebl, 2014; Proverbio et al., 2014, 2015; Pantev et al., 2015; Møller et al., 2018; Sorati and Behne, 2020; Wilbiks and O’Brien, 2020). While integration across modalities appears less prominent in experts who may be better at segregating auditory features from audiovisual compounds (Møller et al., 2018, 2021), our study suggests that feature integration within the relevant modality may simultaneously increase with training.

Consistent with a causal interpretation of our data in the context of the integrated processing hypothesis, decreased neural activity is observed for perceptual learning in audition (Jäncke et al., 2001; Berkowitz and Ansari, 2010; Zatorre et al., 2012) and vision (Schiltz et al., 1999; van Turennout et al., 2000; Kourtzi et al., 2005; Yotsumoto et al., 2008). These changes are sometimes associated with enhanced effective connectivity between distinct cortical regions (Büchel et al., 1999). Enhanced connectivity in auditory areas may also enable musicians to rely more heavily on auditory than visual information in audiovisual tasks (Paraskevopoulos et al., 2015; Møller et al., 2018). This seems adaptive assuming that auditory information is better integrated and thus potentially more informative and relevant to musicians. Correspondingly, greater cortical thickness correlation between visual and auditory areas have been found in non-musicians compared to musicians (Møller et al., 2021).

Potentially more integrative auditory processing may indeed benefit musicians behaviorally. For instance, this may manifest in terms of enhanced verbal and visual memory (Chan et al., 1998; Jakobson et al., 2008). This would be consistent with feature-based theories of visual short-term memory positing that integrative processing allows experts to incorporate multiple features into object representations, thus improving discrimination of highly similar exemplars (Curby et al., 2009).

### Frequency-related feature selectivity

Whereas MMNm subadditivity was present for double and triple deviants comprising frequency, intensity, and location, expertise only interacted with additivity for frequency-related deviants. This aligns well with the different levels of musical relevance embodied by these features, with frequency, intensity, and location representing decreasingly common parameters of syntactic organization in music. More specifically, frequency is usually predetermined by composers in terms of unambiguous notation of intended pitch categories, the spontaneous changing of which would produce distinctly different melodies. Intensity is subject to more flexible expressive performance decisions (Palmer, 1996; Widmer and Goebl, 2004), and changing it would usually not compromise syntax or melodic identity. Sound source localization is relatively unimportant in the professional lives of musicians and non-musicians, with the possible exception of orchestral and choral conductors whose more advanced sound localization skills are evident from specialized neuroplasticity (Münte et al., 2001; Nager et al., 2003). Given that our musician group, however, only comprised a single individual with conducting experience, we can assume that all members of this group had intensive pitch-related training, that most members regularly used intensity as an expressive means, but that sound source localization was overall of secondary importance.

The prominence of pitch over intensity and location may relate to the diverging potential for learning effects to emerge within these features. For example, while learning of frequency discrimination is ubiquitous (Kishon-Rabin et al., 2001), adaptation to altered sound-localization cues is only partial in the horizontal plane (Hofman et al., 2002; Wright and Zhang, 2006). Indeed, recognizing and producing pitch accurately is rehearsed intensively in musical practice (Besson et al., 2007), and voice pedagogues regard pitch intonation as the most important factor in determining singing talent (Watts et al., 2002). Even beyond musical contexts, musicians up-weight pitch compared to duration when determining linguistic focus in speech (Symons and Tierney, 2021).

One possible interpretation following from the present study is that musical learning may involve changes in feature integration which, accordingly, would manifest most clearly in the musically relevant and perceptually plastic pitch-related domain. Indeed, musical pitch information has been shown to be integrated with intensity (Grau and Kemler-Nelson, 1988; McBeath and Neuhoff, 2002) and timbre (Krumhansl and Iverson, 1992; Hall et al., 2000; Lidji et al., 2009; Allen and Oxenham, 2014), at least in parietal cortex (Sohoglu et al., 2020), manifesting as nearly complete spatial overlap in neural processing of frequency and timbre at the inter-subject level (Allen et al., 2017). More broadly, the three features included here exemplify both the “what” (frequency, intensity) and “where” (location, intensity) streams of auditory processing, which have been found to follow different patterns of feature integration (Delogu et al., 2014).^3^ By mostly recruiting participants with uniform expertise levels, many previous studies have avoided systematic group comparisons capable of demonstrating expertise-related differences in feature integration.

In sum, although frequency, intensity, and location all have relevance in music, frequency represents the most salient cue for identifying and distinguishing musical pieces and their component themes and motifs. Therefore, when musicians show selectively affected frequency processing, we infer this to be more likely due to their intensive training than to innate predispositions (Hyde et al., 2009; Herdener et al., 2010). Even if aspects of innately enhanced frequency-specific processing are present, they are likely to require reinforcement through intensive training, possibly motivated by greater success experiences as a young musician. While these learning effects may transfer to prosodic production and decoding (Besson et al., 2007; Pastuszek-Lipińska, 2008; Lima and Castro, 2011), they are of limited use outside musical contexts where sudden changes in intensity or location are usually more likely than frequency to constitute environmentally relevant cues.

### Context-dependency of expert feature processing

It is plausible that because superior frequency processing is merely adaptive in relevant contexts, musicians showed greater frequency-related feature integration for more complex, musically relevant stimuli. This corresponds with stronger MMNm amplitudes, shorter MMNm latencies, and more widespread cortical activation observed in musically complex compared to simple oddball paradigms (Lappe et al., 2016). Crucially, “complexity” in this regard refers to degrees of musical relevance (i.e., ecological validity) whereas increasing the statistical complexity of the context in which deviants are presented—for example, in terms of entropy—attenuates mismatch responses for both musicians and non-musicians (Quiroga-Martinez et al., 2020). Similarly, greater MMNm amplitudes and shorter latencies typically emerge for spectrally rich tones (e.g., realistic piano tones) compared to pure tones that only rarely occur naturally (Tervaniemi et al., 2000). Musical expertise, moreover, produces greater MMNm amplitudes (Fujioka et al., 2004) and more widespread activation (Pantev et al., 1998) for complex than for pure tones. Enhanced processing of audiovisual asynchronies in musicians is also restricted to music-related tasks, remaining absent for language (Lee and Noppeney, 2014). Research in other expertise domains has found comparable levels of domain-specificity (de Groot, 1978; Ericsson and Towne, 2010).

Consistent with the context-specificity demonstrated here, we have previously found in behavioral experiments that musical stimuli activate more specific predictions in musicians and thus evoke stronger reactions when expectations are violated (Hansen and Pearce, 2014; Hansen et al., 2016). In what may first appear to diverge from the current results, this previous work showed that expertise effects were sometimes only detectable in stylistically simple conditions (Hansen and Pearce, 2014). The notion of complexity does, however, not directly translate to the current study in that the simple melodies used by Hansen and Pearce (2014) were always substantially more complex than the complex stimuli in the current MEG paradigm. The psychological processes under investigation were also much higher level (i.e., conscious expectations for discrete pitches within a scale vs. pre-attentive, early neural responses to mistuned pitches) and thus would have relied on (at least partially) distinct neural mechanisms. Because the expertise-by-complexity interaction already disappeared in a more implicit task where listener uncertainty was inferred from probe-tone ratings for multiple melody continuations, we would also not expect this effect to transfer to early neural responses.

Observations that pitch and duration in melodies are integrated when these dimensions co-vary—but not when they contrast—and that such integration increases with exposure (Boltz, 1999) suggest an interpretation of our results where musicians are predisposed to capitalize more optimally upon contextual cues. Note, though, that the two present paradigms did not only differ on musical relevance, but also on stimulus onset asynchrony, predictability of deviant location, and general textural complexity. Although these differences between the two paradigms do not compromise our main finding of expertise-related subadditivity for frequency-related features, future research should try to disentangle the individual contribution of these factors.

### Future research and unresolved issues

Some clinical studies have indeed measured neural responses to multiple co-occurring auditory feature deviants (e.g., Hay et al., 2015) and have found, for example, that MMNs from double deviants combining duration and frequency–but not their constitutive single-deviant MMNs–predict the time to psychosis onset in individuals at high risk for schizophrenia (Perez et al., 2014; Hamilton et al., 2022). These studies have, however, not typically assessed MMN(m) additivity *per se*. Thus, there may be an underused potential for extending the methods and results applied and developed here to relevant clinical populations in future work.

It is worth noting that the increased frequency-related feature integration in musicians observed here seems inconsistent with other behavioral and neuroimaging results. For example, a behavioral study found that interference of pitch and timbre was unaffected by expertise (Allen and Oxenham, 2014). Conversely, findings that pitch and consonants in speech produce sub-additive MMN responses (Gao et al., 2012), but do not interact behaviorally (Kolinsky et al., 2009), suggest that measures of behavioral and neural integration do not always correspond (Musacchia et al., 2007). Additionally, cross-modal integration often results in increased rather than decreased neural activity (Stein, 2012). For instance, trumpeters’ responses to sounds and tactile lip stimulation exceed the sum of constituent unimodal responses (Schulz et al., 2003). When reading musical notation, musicians dissociate processing of spatial pitch in the dorsal visual stream from temporal movement preparation in the ventral visual stream (Bengtsson and Ullén, 2006) and show no neurophysiological or behavioral interference of frequency and duration (Schön and Besson, 2002). This would suggest greater feature independence in experts.

The Reverse Hierarchy Theory of perceptual learning supports this view by asserting that expertise entails more segregated representations of ecologically relevant stimuli, thus providing access to more detailed lower-level representations (Ahissar and Hochstein, 2004; Ahissar et al., 2009). This has some bearing on musical intuitions. Specifically, segregated processing would presumably better enable musicians to capitalize upon the covariance of acoustic features, as demonstrated for tonal and metrical hierarchies (Prince and Schmuckler, 2014). Phenomenal accents in music are indeed intensified by coinciding cues (Lerdahl and Jackendoff, 1983; Hansen, 2011), which may underlie musicians’ superiority in decoding emotion from speech (Lima and Castro, 2011) and segmenting speech (François et al., 2013, 2014) and music (Deliège, 1987; Peebles, 2011, 2012). Given that individuals with better frequency discrimination thresholds more capably segregate auditory features from audiovisual stimuli (Møller et al., 2018), it remains unknown whether musicians suppress frequency-related feature integration whenever constituent features are irrelevant to the task at hand. The interaction of expertise with frequency-related feature-selectivity and context-specificity observed here contributes crucial empirical data to ongoing discussions of seemingly diverging findings pertaining to integrated and independent musical feature processing (Palmer and Krumhansl, 1987; Boltz, 1999; Waters and Underwood, 1999; Tillmann and Lebrun-Guillaud, 2006).

## Data availability statement

To the extent that local ethical and legal regulations allow, the data supporting the conclusions of this article will be made available by the authors, without undue reservation.

## Ethics statement

The studies involving human participants were reviewed and approved by The Central Denmark Regional Committee on Health Research Ethics (case 1-10-72-11-15). The participants provided their written informed consent to participate in this study.

## Author contributions

NH: conceptualization, methodology, software, validation, formal analysis, investigation, data curation, writing—original draft and review and editing, visualization, project administration, and funding acquisition. AH: conceptualization, methodology, software, validation, formal analysis, investigation, writing—review and editing, and supervision. CM: conceptualization, methodology, investigation, and writing—review and editing. MP: conceptualization, writing—review and editing, and supervision. PV: conceptualization, resources, writing—review and editing, supervision, and funding acquisition. All authors contributed to the article and approved the submitted version.

## Footnotes

1
http://www.ru.nl/neuroimaging/fieldtrip

2
http://www.fieldtriptoolbox.org/faq/how_can_i_test_an_interaction_effect_using_cluster-based_permutation_tests

3 The coupling of frequency and intensity cues for determining the identity of auditory objects may seem obvious. With regards to the coupling of intensity and location cues, research by Hansen and Huron (2019), for example, demonstrates how manipulating intensity can alter perception of location and motion in physical space.
