# Supplementary material for: Musicians show more integrated neural processing of contextually relevant acoustic features
Source: Front Neurosci. 2022 Oct 13;16:907540. doi: 10.3389/fnins.2022.907540 (PMC9612920; doi:10.3389/fnins.2022.907540)
Supplement: Supplementary file 1 [file Data_Sheet_1.docx]

Supplementary Material

|  | **Complex musical multi-feature paradigm** | |
| --- | --- | --- |
|  | Musicians | Non-musicians |
| FI | 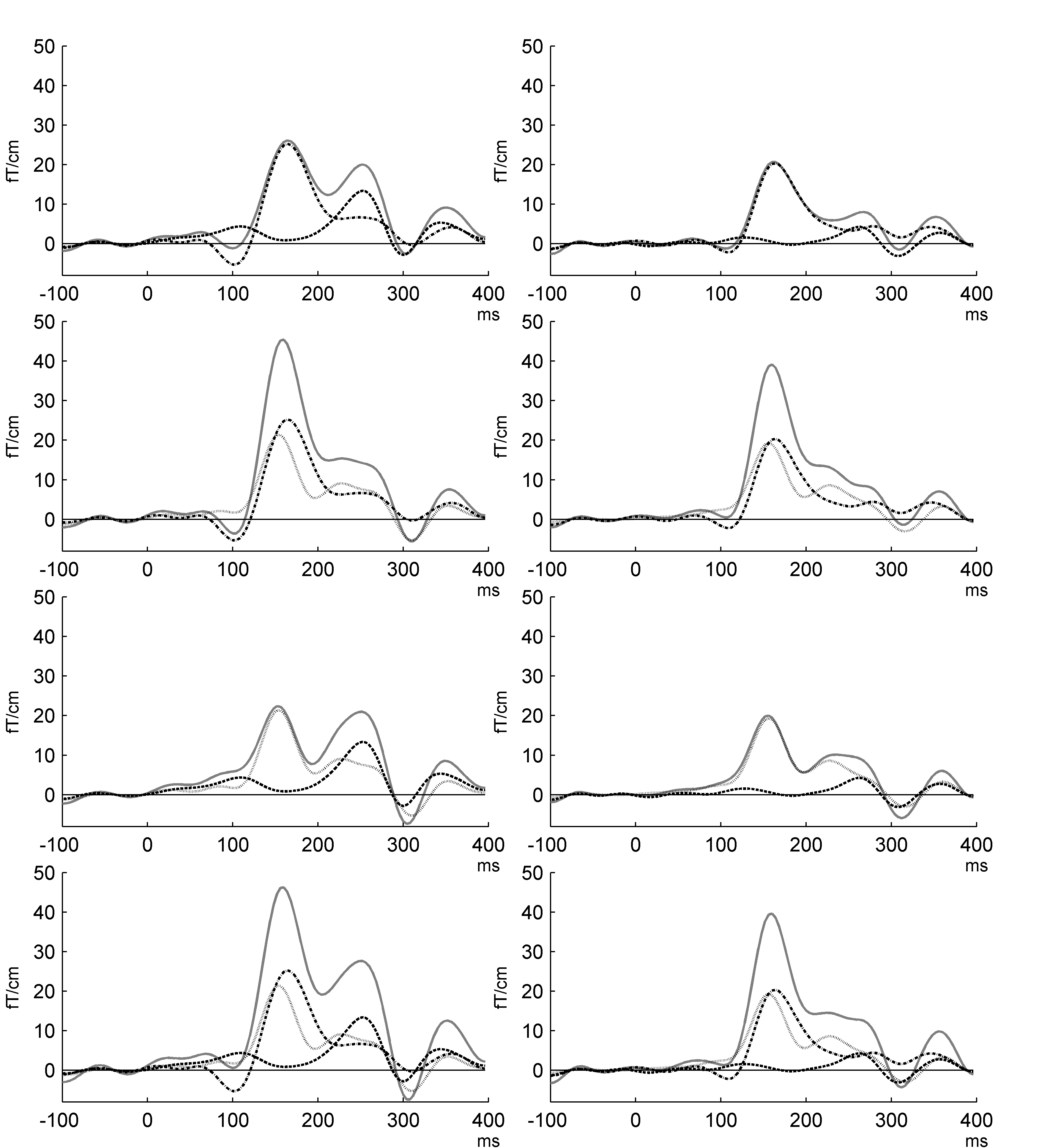 | |
| IL |  |  |
| LF |  |  |
| FIL |  |  |
| 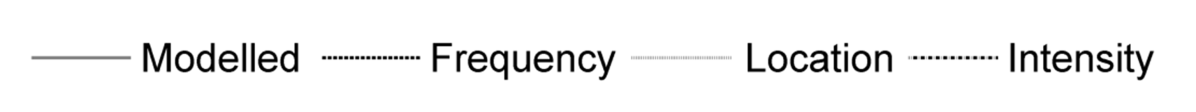 | | |

**Supplementary Figure 1.** **Modelled MMNm responses in the complex musical multi-feature paradigm**. Plots of the MMNm for individual single-feature deviants and the modelled MMNm response resulting from summation of these responses.

|  | **Simple control paradigm** | |
| --- | --- | --- |
|  | Musicians | Non-musicians |
| FI | 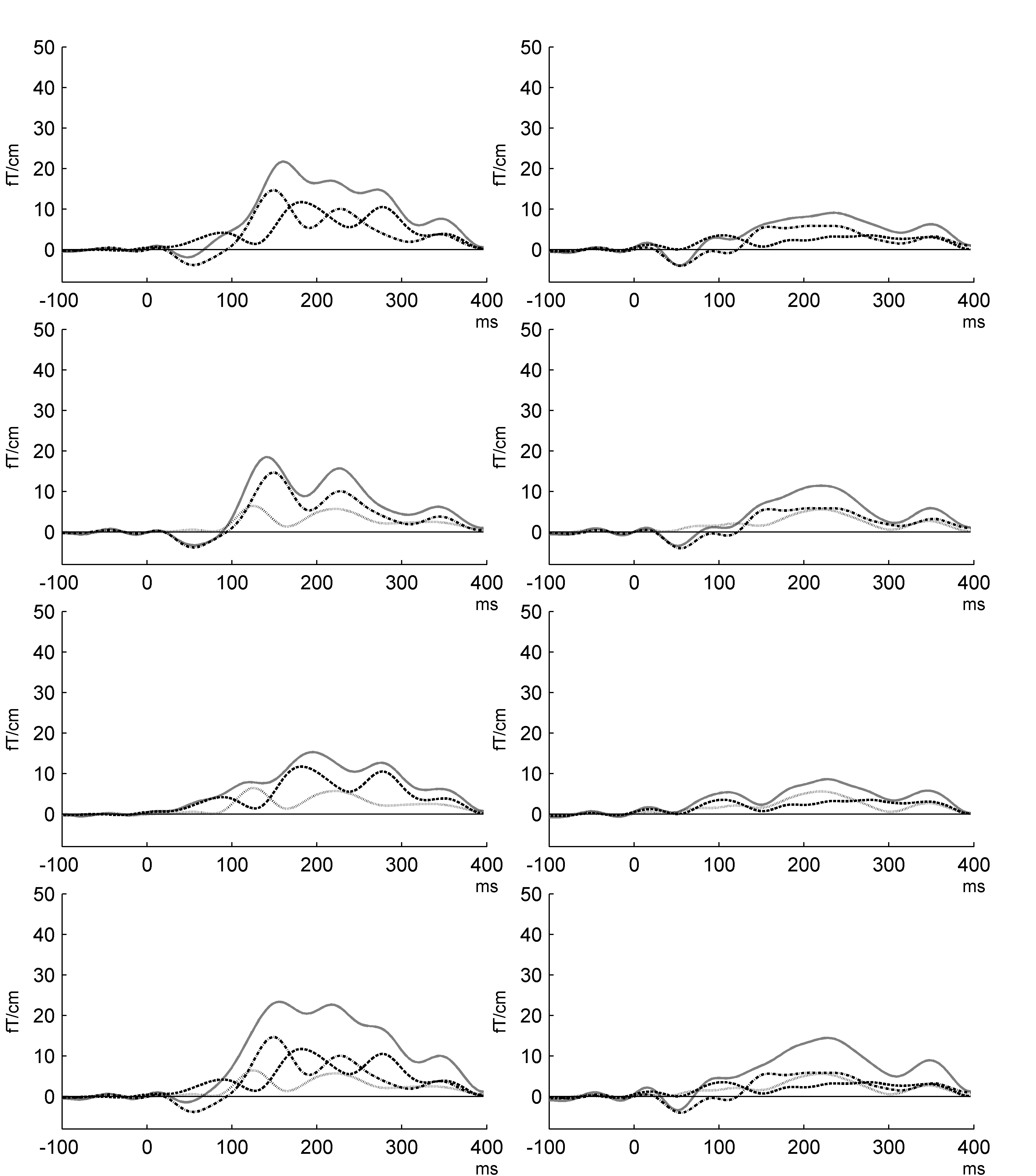 | |
| IL |  |  |
| LF |  |  |
| FIL |  |  |
| 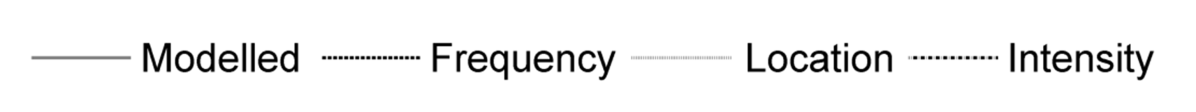 | | |

**Supplementary Figure 2.** **Modelled MMNm responses in the simple control paradigm**. Plots of the MMNm for individual single-feature deviants and the modelled MMNm response resulting from summation of these responses.
